# Effects of liposoluble components of highland barley spent grains on physiological indexes, intestinal microorganisms, and the liver transcriptome in mice fed a high‐fat diet

**DOI:** 10.1002/fsn3.3291

**Published:** 2023-04-21

**Authors:** Jiali Zhang, Yihao Luo, Shengbao Feng, Wancheng Sun, Shanwen Li, Lingwu Kong

**Affiliations:** ^1^ College of Agriculture and Animal Husbandry Qinghai University Qinghai China; ^2^ Qinghai Huzhu TianYouDe Highland Barley Spirit Co., Ltd. Qinghai China

**Keywords:** highland barley spent grains, intestinal flora, lipid metabolism, transcriptomics

## Abstract

The purpose of this study was to investigate the effects of the active ingredients of barley lees on the physiological indexes, intestinal flora, and liver transcriptome of mice fed a high‐fat diet. Twenty‐four male C57BL/6J mice were randomly divided into 4 groups and fed the experimental diets for 5 weeks. The results showed that the fat‐soluble components of distillers' grains significantly reduced body weight, abdominal fat, perirenal fat, blood glucose, low‐density lipoprotein cholesterol, triglycerides, and total cholesterol in the high‐fat diet‐fed mice (*p* < .05), significantly decreased alanine aminotransferase and malondialdehyde levels, and significantly increased total superoxide dismutase, catalase, reduced glutathione and glutathione peroxidase levels (*p* < .05). At the phylum level, lipid‐soluble components significantly increased the abundance of *Bacteroidetes* and decreased the *Firmicutes/Bacteroidetes* ratio. At the genus level, the relative abundances of *Bacteroidetes* and *Clostridium* were increased. Transcriptomic analysis showed that lipid‐soluble components of spent grains reduced the mRNA expression of ANGPTL8, CD36, PLTP, and SOAT1 and increased the mRNA expression of CYP7A1 and ABCA1 in the cholesterol metabolism pathway, promoted the transport of cholesterol, and inhibited the absorption of cholesterol, which can decrease cholesterol levels by speeding up the conversion of cholesterol into bile acids.

## Graphical abstract

The liposoluble components of highland barley grains significantly improved physiological and biochemical indexes and liver antioxidant indexes of mice on high‐fat diet. The liposoluble components of highland barley grains significantly changed the intestinal microflora diversity and community structure of high‐fat diet mice. The liposoluble components of highland barley grains significantly improved cholesterol metabolism pathway in high‐fat diet mice by regulating the expression of ANGPTL8, CD36, PLTP, SOAT1, CYP7A1, and ABCA1 genes.

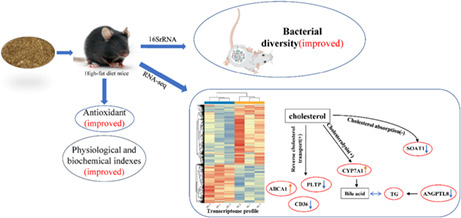

## 1 INTRODUCTION

In recent years, due to the rapid development of human society and the rapid improvement of people's living standards, obesity has become the fifth leading cause of death in the world and the third leading epidemiological factor affecting human health after smoking and AIDS (Smith & Smith, 2016). The occurrence of obesity is closely related to lipid metabolism disorders. When the body's energy intake and expenditure are unbalanced, adipose tissue stores excess nutrients in the form of triglycerides, resulting in excessive increases in the number of adipocytes and their cell volume and even apoptosis or necrosis of adipocytes, leading to the pathological state of excessive accumulation and/or abnormal distribution of fat (Hu et al., 2021). Studies have shown that lipid metabolism disorders cause increases in BMI, total cholesterol (T‐CHO), total triglycerides (TG), and other biochemical indicators used to judge obesity and hyperlipidemia, and if not controlled, they increase the risks of diabetes, nonalcoholic fatty liver disease, aortic atherosclerosis, and other diseases (Chen et al., 2018).

The intestinal flora plays an important role in the physiological processes of the host, such as the digestion and absorption of nutrients, utilization and storage of energy and metabolism. It is an “organ” containing bacteria that are mainly distributed in the colon (Lee et al., 2020). The colonization pattern of microorganisms in the intestine plays a key role in health and serves as a unique biomarker for identifying the physiological state of each individual (Villanueva‐Millán et al., 2015). Obesity alters the composition and relative abundance of intestinal microorganisms. The structure of the intestinal flora, such as the proportions of *Firmicutes* and *Bacteroides* and the abundance of beneficial bacteria, and the changes in intestinal diversity comprehensively affect lipid metabolism (Junior et al., 2021).

The transcriptomic analysis technology of RNA sequencing (RNA‐seq) is a powerful tool that has become indispensable for analyzing differential gene expression and clarifying the potential mechanisms of various complex diseases within the scope of omics research (Gao et al., 2020). RNA‐seq technology has been widely used to study the mechanisms of action of various active components of disease resistance. Hou et al. (2021) found that mungbean can alleviate liver steatosis in obese mice fed a high‐fat diet and observed enrichment of the NOD‐like receptor signaling pathway and Toll‐like receptor signaling pathway in KEGG pathway analysis; these data laid foundation for explaining the mechanism by which mungbean alleviates liver steatosis in obese mice. Zhu et al. (2019) and others used RNA‐seq technology to sequence the liver transcriptome of rats with diet‐induced obesity treated with patchouli and found that the lipid‐lowering effect of patchouli on rats with diet‐induced obesity may be mediated by regulating the expression of Fasn‐, Socs2‐, and Ppp1r3b‐related genes and proteins in the insulin signaling pathway.

Highland barley spent grains are a byproduct of the fermentation of highland barley BAIJIU, and the yield of highland barley spent grains is high. In addition, highland barley spent grains are rich in nutrients including protein, fat, crude fiber *β*‐glucan, B vitamins, and phenols and are potential sources of many bioactive components; however, the utilization of highland barley spent grains has always been a low‐value option, resulting in great resource waste and environmental pollution (Li et al., 2018). As people pay increasing attention to their dietary structure and health, research on the active components of natural products and their physiological functions and mechanisms of action has become important in the fields of modern food and nutrition science. It has been found that Baijiu distiller's lees (Luo, Xi, et al., 2022) have bacteriostasis (Wang et al., 2016), antitumor, anticancer (Wang et al., 2021), and other properties, but there are few studies on the active ingredients of barley lees. In addition, our previous study found that the liposoluble components of highland barley lees were rich in various unsaturated fatty acids, sterols, and other active ingredients, among which the unsaturated fatty acid content was 67.09%, the monounsaturated fatty acid content was 19.66%, the polyunsaturated fatty acid content was 47.43%, and the content of β‐sitosterol in phytosterols was 52.78% (Zhang et al., 2023). These active ingredients play an important physiological role in a variety of diseases; for example, fatty acids can prevent inflammation and cancer, regulate blood pressure and lipids, and prevent cardiovascular and cerebrovascular diseases, and plant sterols can reduce cholesterol, inhibit tumors, and regulate immunity and, most importantly, the analysis and application of the active components of barley lees in lipid metabolism, the differentially dominant components of the intestinal flora and differentially expressed genes at the transcriptome level have not been reported. Therefore, the effects of the active components of barley lees on lipid metabolism disorders, the intestinal flora and the transcriptome in mice fed a high‐fat diet were investigated. The results provide a theoretical basis for the development and utilization of barley lees.

## 2 MATERIALS AND METHODS

### 2.1 Animals, materials, and reagents

The experimental animals were 4‐week‐old male SPF‐grade C57BL/6J mice with body weights of approximately 16.00 g, and the production license number for the experimental animals was SCXK (Shaanxi) 2018–001. The mice were fed and harvested according to order SL‐2021019 of the Qinghai University Ethics Committee.

The experimental feed consisted of low‐fat feed (TP23302, fat content of 10%) or high‐fat feed (TP23300, fat content of 60%), purchased from Trophic Animal Feed High‐Tech Co., Ltd, China.

The liposoluble components of highland barley spent grains were produced in our laboratory. The preparation methods were as follows: remove impurities from highland barley dry spent grains and crush them with a high‐speed crusher. Then, accurately weigh the crushed highland barley spent grain powder and load it into the extraction kettle, and set the temperature of the extraction kettle and separation kettle. After constant temperature is reached, start the high‐pressure pump to inject CO_2_, adjust the pressure of the extraction kettle to the set level and start the timer. After extraction, the liposoluble components are collected from the separation kettle.

The main reagents used were total cholesterol, triglycerides, high‐density lipoprotein cholesterol, low‐density lipoprotein cholesterol, fasting blood glucose, alanine aminotransferase, and aspartate transaminase, purchased from Shenzhen Rayto Life Sciences Co., Ltd. The test boxes for the determination of total superoxide dismutase, malondialdehyde, glutathione peroxidase, catalase, and reduced glutathione were purchased from Nanjing Jiancheng Bioengineering Research Institute Co., Ltd.

### 2.2 Experimental methods

#### 2.2.1 Establishment and grouping of animal models

Twenty‐four male C57BL/6J mice were randomly divided into 4 groups with 6 mice in each group after adaptive feeding for one week. The specific groups of mice were as follows: low‐fat group (LF group), high‐fat group (HF group), positive control group (PC group, orlistat added at a rate of 1.20%), and experimental group (SE group, spent grain liposoluble components added at a rate of 1.20%). While the mice in the low‐fat group were fed low‐fat feed, the mice in the three other groups were fed high‐fat feed, high‐fat feed + orlistat, and high‐fat feed + spent grain liposoluble components, respectively. All mice were placed under conditions of constant temperature and humidity with a 12‐h dark cycle and free access to food and drinking water. The feed was topped up every day, and the food intake was measured, and the body weight was measured every week. After feeding the mice the liposoluble components of spent grains for 4 weeks, they were fasted for 12 h and then anesthetized; thereafter, their eyes were removed, their blood was taken, and the mice were killed. The mice were weighed before death. After anesthetization and blood collection, the abdominal cavity and chest cavity of the mice were opened, and the heart, liver, spleen, kidney, abdominal fat, and perirenal fat were immediately separated and weighed. After weighing, the abdominal fat and 100 mg of liver tissue were fixed with 4% paraformaldehyde for histopathological observation. The remaining tissues were stored at −80°C for later use.

#### 2.2.2 Calculation of the body weight gain, organ index, abdominal fat index, and perirenal fat index of mice

On the day before the beginning of the experiment, the mice in each group that had been fasted for 12 h were weighed on an empty stomach to obtain the initial body weight. Before the mice were killed, the fasting weight of mice that had been fasted for 12 h was recorded as the final weight. Finally, the weight change increment of the mice was calculated. After the mice were killed, their intact organs, abdominal fat, and perirenal fat were removed, and the corresponding indexes were calculated.

$$\text{Index of each component}=\frac{\text{mass of each component}\;\left(\mathrm{mg}\right)}{\text{body weight}\;\left(g\right)}$$

#### 2.2.3 Determination of serum and liver biochemical indexes

Mouse eye blood was collected with a 2‐mL sterile EP tube. After standing at room temperature for 1 h, the blood was centrifuged at 4°C and 2264 *g* for 10 min. The upper serum was absorbed, subpacked, and stored at −80°C. The concentrations of total cholesterol, triglycerides, high‐density lipoprotein cholesterol, low‐density lipoprotein cholesterol, and glucose in the serum were measured according to the kit method. The levels of alanine aminotransferase and aspartate aminotransferase in liver tissue were also measured according to the kit method.

#### 2.2.4 Liver antioxidant index determination

An appropriate amount of liver tissue was weighed and ground with 9 times the amount of homogenate medium. Then, the grinding liquid was centrifuged at 3000–4000 r for 10 min, and the supernatant was taken to prepare 10% tissue homogenate. The antioxidant indexes of the liver were determined according to the kit method. The determined antioxidant indexes included those of total superoxide dismutase, malondialdehyde, glutathione peroxidase, catalase, and microreduced glutathione.

#### 2.2.5 Pathological analysis of liver and adipose tissue

Liver and abdominal adipose tissues were collected and fixed with 4% paraformaldehyde. Paraffin sections were stained with hematoxylin and eosin (H&E; Luo, Sun, et al., 2022). Histopathological changes were observed under an optical microscope, and images were recorded.

#### 2.2.6 Flora analysis of mouse intestinal contents

After the mice were sacrificed, their cecal contents were collected using a sterile EP tube. A QIAamp‐DNA stool mini kit was used to extract DNA from colon content specimens, and a variable region of 16 S rDNA was amplified. The Illumina NovaSeq platform was used for double‐ended sequencing of the sequencing samples. Data analysis was carried out on the Wekemo Bioincloud.

#### 2.2.7 Transcriptomic analysis of mouse liver

##### RNA extraction and transcriptome sequencing

RNA was extracted from tissues or cells via a standard extraction method, and the RNA samples were then subjected to strict quality control. An Agilent 2100 Bioanalyzer was used for quality control to accurately assess the integrity of the RNA. Because most eukaryotic mRNAs contain poly‐A bases, mRNAs with poly‐A bases were enriched by magnetic beads connected with poly‐T bases. Then, the obtained mRNA was randomly interrupted with divalent cations in NEB fragmentation buffer, and the library was built according to the NEB common library building method. After the library was constructed, Qubit 2.0 was used as the first fluorometer for preliminary quantification, the library was diluted to 1.5 ng/μL, and an Agilent 2100 Bioanalyzer was then used to determine the insert size of the library. After the insert size met the expectation, qRT‐PCR was performed to accurately quantify the effective concentration of the library (the effective concentration of the library was higher than 2 nm) to ensure the quality of the library. After passing the library inspection, different libraries were pooled according to the required effective concentration and target offline data volume, and Illumina sequencing was then carried out. The basic principle of sequencing was sequencing by synthesis. Four fluorescently labeled dNTP, DNA polymerase, and linker primers were added to the sequenced flow cell for amplification. When each sequencing cluster extended the complementary chain, each fluorescently labeled dNTP released the corresponding fluorophore. The sequencer captured the fluorescence signal and converted the optical signal into a sequencing peak with computer software to obtain the sequence information of the fragment to be tested (He et al., 2018).

##### Transcriptomic data analysis

Clean data were obtained after the raw data obtained by sequencing were subjected to quality control. The mapped reads were spliced and compared with the annotation information for the reference genome. In this study, the fragments per kb per million reads (FPKM) method was used to calculate gene expression levels. The differentially expressed genes were screened with the DEseq2 software package. The screening thresholds were a fold change ≥ l.5 and a *p* value < .05. The R language software package clusterProfiler was used for Gene Ontology (GO) functional annotation and Kyoto Encyclopedia of Genes and Genomes (KEGG) pathway enrichment analysis of differentially expressed genes.

#### 2.2.8 Data processing

The experimental data were statistically analyzed by Prism software. Comparisons between multiple groups were analyzed by one‐way analysis of variance (ANOVA). The data were expressed as the mean ± standard error, and the differences were statistically significant at *p* < .05.

## 3 RESULTS

### 3.1 Effect of liposoluble components of spent grains on weight change in mice

**FIGURE 1:**
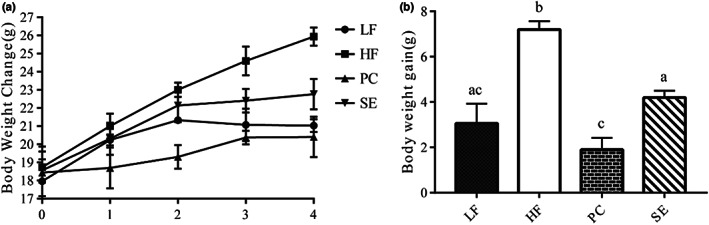
Effects of liposoluble components in highland barley grains on body weight change and increment in mice (a) body weight change diagram; (b) weight gain diagram.

Compared with the LF group, the HF group showed rapid weight growth and a significant difference in weight gain (*p* < .05; Figure 1). Compared with the HF group, the body weight increments in the PC group and SE group were significantly decreased (*p* < .05). The above results show that the effects of SE on body weight and body weight gain are similar to those of LF and PC. Thus, the liposoluble components of spent grains have a good effect on the control of body weight gain.

### 3.2 Effects of liposoluble components of spent grains on the organ index and abdominal and perirenal fat index in mice fed a high‐fat diet

Compared with the LF group, the heart, kidney, liver and spleen indexes of mice in the HF group were significantly higher (*p* < .05) (Table 1). Compared with the HF group, the spleen, heart, liver, and kidney indexes of the mice in each other group were lower, and the spleen and kidney indexes of the mice in the SE group were significantly decreased by 14.02% and 7.43% (*p* < .05), respectively. In addition, the abdominal fat and perirenal fat indexes of the HF group were significantly higher than those of the LF group (*p* < .05), and the abdominal fat and perirenal fat indexes of the SE group were significantly lower than those of the HF group (*p* < .05). The above results showed that the liposoluble components of spent grains had no significant effect on the heart or liver of the mice and could improve the changes in the kidney and spleen indexes and alleviate the accumulation of abdominal fat and perirenal fat induced by a high‐fat diet in mice.

**TABLE 1:** Organ index, abdominal index, and perirenal index of mice in each group (X ± SD, *n* = 6).

Groups	Liver index (mg/g)	Cardiac index (mg/g)	Spleen index (mg/g)	Kidney index (mg/g)	Abdominal fat index (mg/g)	Perirenal fat index (mg/g)
LF	32.66 ± 0.51^a^	5.32 ± 0.18^a^	3.26 ± 0.22^a^	12.74 ± 0.96^a^	6.16 ± 0.90^a^	0.88 ± 0.03^a^
HF	37.04 ± 1.13^c^	6.58 ± 0.30^c^	3.85 ± 0.04^c^	15.88 ± 0.45^b^	46.43 ± 2.82^b^	1.54 ± 0.03^b^
PC	31.17 ± 0.59^a^	4.92 ± 0.013^a^	2.66 ± 0.16^b^	11.75 ± 0.32^c^	12.30 ± 1.88^a^	0.98 ± 0.05^c^
SE	36.56 ± 0.23^bc^	6.26 ± 0.36^bc^	3.31 ± 0.12^a^	14.70 ± 0.44^d^	24.91 ± 3.56^c^	0.95 ± 0.02^ac^

Different letters demonstrate significance at *p* < .05.

### 3.3 Effects of liposoluble components of spent grains on serum lipid levels in mice fed a high‐fat diet

Compared with the LF group, the levels of T‐CHO, TG, LDL‐C and GLU in the HF group were increased significantly, and the level of HDL‐C was decreased significantly (*p* < .05). Compared with the HF group, the levels of T‐CHO, TG, LDL‐C, and GLU in the SE group were decreased significantly (*p* < .05), and there was no significant difference in HDL‐C levels, but there was still a significant increase trend (Table 2).

**TABLE 2:** Effects of spent grains' liposoluble components on serum lipid levels in high‐fat diet mice.

Groups	T‐CHO (mmol/L)	TG (mmol/L)	LDL‐C (mmol/L)	HDL‐C (mmol/L)	GLU (mmol/L)
LF	3.73 ± 0.23^a^	1.76 ± 0.06^a^	0.46 ± 0.04^a^	1.99 ± 0.03^a^	3.27 ± 0.58^a^
HF	5.12 ± 0.14^b^	3.01 ± 0.02^b^	1.35 ± 0.09^b^	1.55 ± 0.17^b^	7.63 ± 0.21^b^
PC	3.84 ± 0.50^a^	2.18 ± 0.15^a^	0.53 ± 0.04^a^	1.96 ± 0.04^ac^	3.39 ± 0.55^a^
SE	3.94 ± 0.08^a^	2.00 ± 0.24^a^	0.81 ± 0.06^c^	1.64 ± 0.16^bc^	4.36 ± 0.20^a^

Different letters demonstrate significance at *p* < .05.

### 3.4 Effects of spent grain liposoluble components on the liver indexes of high‐fat diet‐fed mice

Compared with the LF group, the high‐fat diet caused a significant increase in liver ALT and AST levels (*p* < .05). Compared with the HF group, the levels of ALT and AST in the livers of the PC group mice were significantly lower (*p* < .05). The levels of ALT in the livers of the SE group mice were significantly lower (*p* < .05), and the level of AST was not statistically significant, but there was still a significant decrease. Moreover, the liposoluble components of spent grains significantly reduced the levels of ALT and AST. The liposoluble components had a significant effect on the antioxidant indexes of SOD, CAT, GSH, GSH‐Px, and MDA in the mouse livers (*p* < .05). Compared with the LF group, the levels of SOD, CAT, GSH, and GSH‐Px in the liver decreased significantly (*p* < .05), and the level of MDA increased significantly (*p* < .05). Compared with the HF group, the levels of SOD, CAT, GSH, and GSH‐Px in the livers of the SE group mice increased significantly (*p* < .05), and the level of MDA decreased significantly (*p* < .05; Table 3).

**TABLE 3:** Effects of liposoluble components of highland barley grains on liver indexes of mice in high‐fat diet.

Index	LF	HF	PC	SE
ALT (U/L)	17.14 ± 0.97^a^	25.75 ± 0.74^b^	18.78 ± 0.59^a^	22.12 ± 0.28^c^
AST (U/L)	13.41 ± 1.24^a^	19.26 ± 0.80^b^	15.43 ± 2.32^a^	16.77 ± 0.47^ab^
SOD activity (U/mgprot)	524.64 ± 22.51^a^	417.64 ± 4.79^b^	483.47 ± 11.14^c^	457.24 ± 8.73^c^
CAT activity (U/mgprot)	59.89 ± 1.05^a^	45.87 ± 0.93^b^	59.88 ± 0.79^a^	52.99 ± 0.82^c^
GSH activity (μmol/gprot)	2.75 ± 0.06^a^	1.84 ± 0.12^b^	2.73 ± 0.15^a^	2.30 ± 0.12^c^
GSH‐Px activity (U/mgprot)	313.37 ± 4.87^a^	229.32 ± 8.64^b^	305.36 ± 10.20^a^	278.37 ± 7.48^c^
MDA activity (nmol/mgprot)	0.54 ± 0.04^a^	1.16 ± 0.04^b^	0.86 ± 0.07^c^	0.83 ± 0.05^c^

Different letters demonstrate significance at *p* < .05.

### 3.5 Effects of liposoluble components of spent grains on the histopathology of liver and abdominal adipose tissue in mice fed a high‐fat diet

**FIGURE 2:**
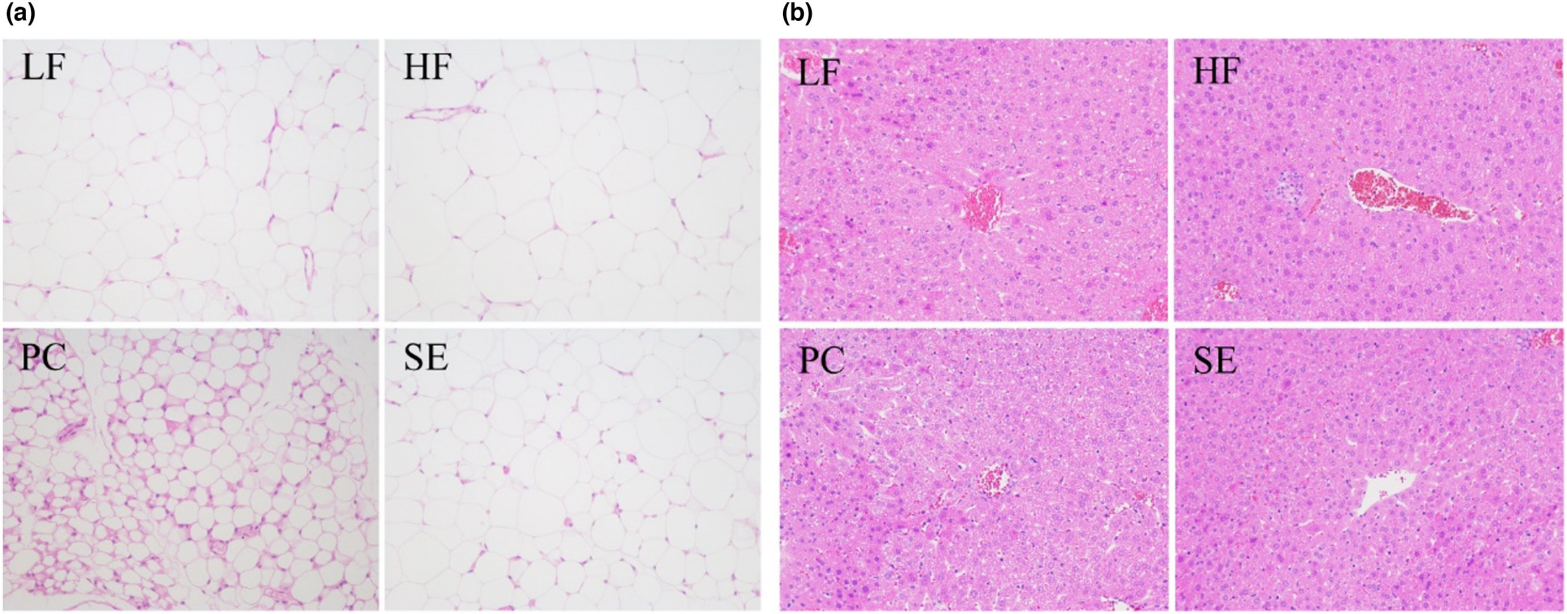
Effects of spent grains' liposoluble components on abdominal fat and liver histopathology in high‐fat diet mice (a) abdominal fat; (b) Liver; magnification ×200.

To study the effect of feeding on spent grain liposoluble components on the pathological changes in liver and abdominal adipose tissue in mice, the liver and abdominal adipose tissue were further stained with H&E. Adipose tissue (Figure 2a): the adipocytes in the LF, PC, and SE groups were tightly packed, uniform in size and round; there was a large lipid droplet in the center of the cell; the cytoplasm was thin, located at the periphery of the cell, and wrapped around the lipid droplet; and the nucleus was oblate and squeezed to one side of the cell by the lipid droplet. In the PC group, a small number of fat cells were disrupted, and their cell shape was irregular. In addition, compared with the HF group, the adipocytes were significantly smaller in the LF group and SE group. The liver tissue (Figure 2b) showed a large amount of hepatocyte steatosis in the LF, PC, and SE groups, tiny circular vacuoles in the cytoplasm, and congestion in the central vein. In the HF group, there was a large amount of hepatocyte steatosis, small circular vacuoles in the cytoplasm, and a small amount of focal infiltration of inflammatory cells in the hepatic lobules. The staining results showed that feeding on the liposoluble components of spent grains could reduce the focal infiltration of inflammatory cells in the liver of mice caused by a high‐fat diet to protect the liver.

### 3.6 Effect of liposoluble components of spent grains on the intestinal flora of mice fed a high‐fat diet

#### 3.6.1 DNA sequence data and operational taxonomy

**FIGURE 3:**
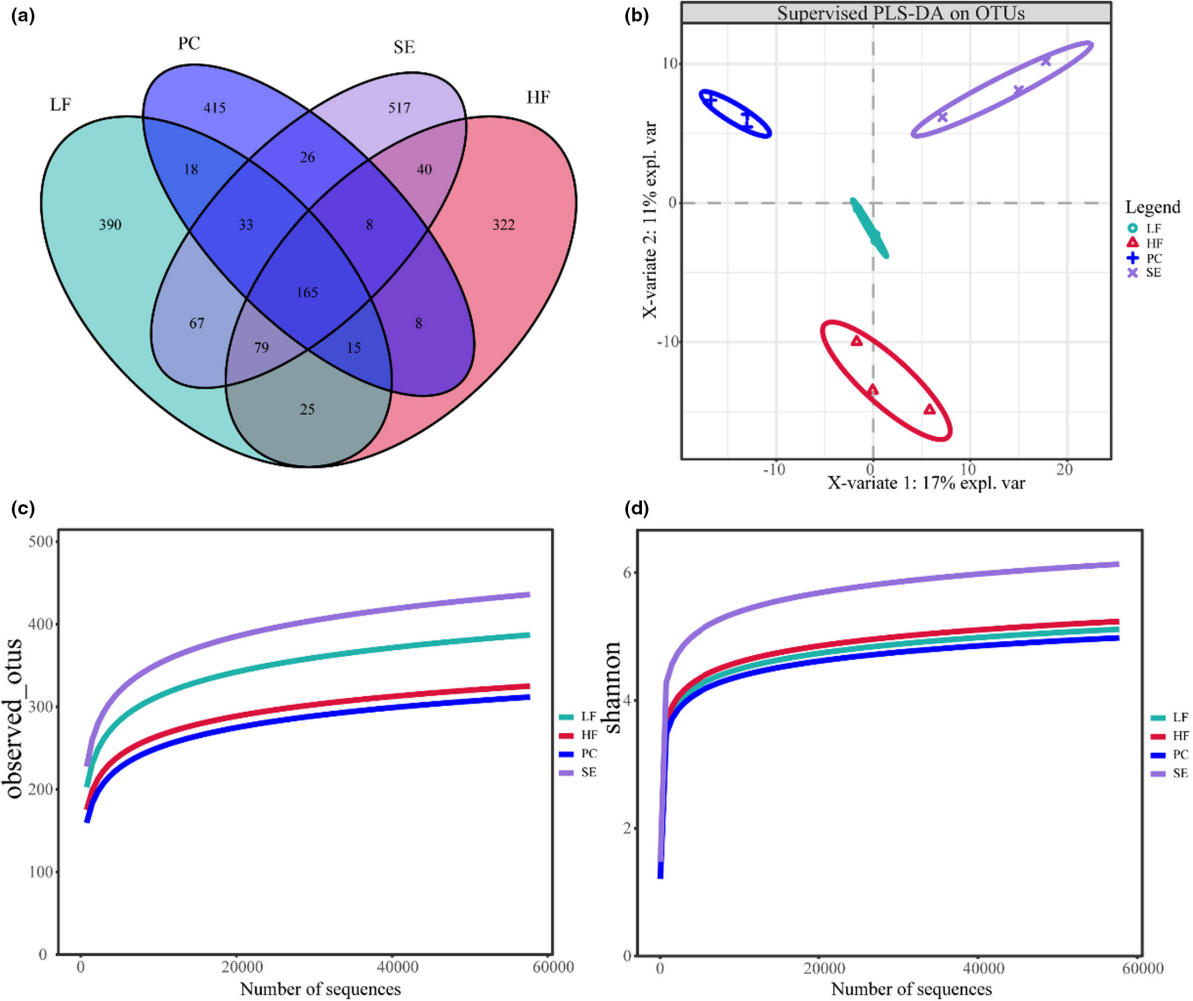
Diversity and composition of intestinal flora of mice in each group (a) OTU distribution Venn diagram. (b) PLS‐DA discriminant analysis. (c) Shannon index curve. (d) Observed_ OTUs index curve.

We generated a Venn diagram to analyze the unique or common OTUs between different sample groups, which was used to count the number of common and unique OTUs in multiple samples, intuitively showing the composition similarity and overlap of samples at the OTU level. After feeding mice the experimental diets for 28 days, the number of unique OTUs in the LF group was 390, that in the HF group was 322, that in the PC group was 415, and that in the SE group was 517. The four groups shared 165 OTUs (Figure 3a).

#### 3.6.2 Analysis of the impact on intestinal flora based on the multivariate discriminant method

A PLS‐DA discriminant model was constructed based on the species abundance matrix, sample grouping data and variable importance in projection (VIP) coefficient of each species. In Figure 3b, the four ellipses represent the four groups, which do not overlap and are clearly separated. The difference between the samples of each group is very small, indicating that species abundance varies greatly between different groups.

#### 3.6.3 Analysis of the alpha diversity of the intestinal flora of mice fed a high‐fat diet with liposoluble components of spent grains

From the alpha diversity dilution curve analysis, it could be seen that the observed OTUs and Shannon curves gradually flattened as the number of sequences continued to increase (Figure 3c,d). These results showed that the obtained sequencing depth was sufficient to reflect the diversity of the test samples. If we were to continue to increase the sequencing depth, we would no longer detect a large number of new OTUs, indicating that the obtained sequencing results were sufficient to reflect the diversity of species contained in the samples and supported the reliability of subsequent analysis. There was no significant difference in the alpha diversity index, but the average value of each index showed an increasing or decreasing trend, indicating that the liposoluble components of highland barley spent grains could increase the richness and diversity of the intestinal flora of mice to a certain extent (Table 4).

**TABLE 4:** Alpha diversity analysis of each group.

Groups	Chao1	Shannon	Simpson	Observed_otus	Faith_pd
LF	395.74 ± 45.10	4.82 ± 1.17	0.84 ± 0.17	395.33 ± 45.08	26.53 ± 7.72
HF	327.80 ± 38.49	4.94 ± 0.80	0.91 ± 0.05	327.00 ± 38.00	25.64 ± 6.58
PC	323.75 ± 35.66	4.70 ± 0.30	0.89 ± 0.03	323.33 ± 36.02	31.03 ± 7.77
SE	449.86 ± 109.15	5.78 ± 0.21	0.95 ± 0.01	449.33 ± 109.00	24.25 ± 1.41

#### 3.6.4 Analysis of the composition and diversity of flora at the phylum and genus levels

**FIGURE 4:**
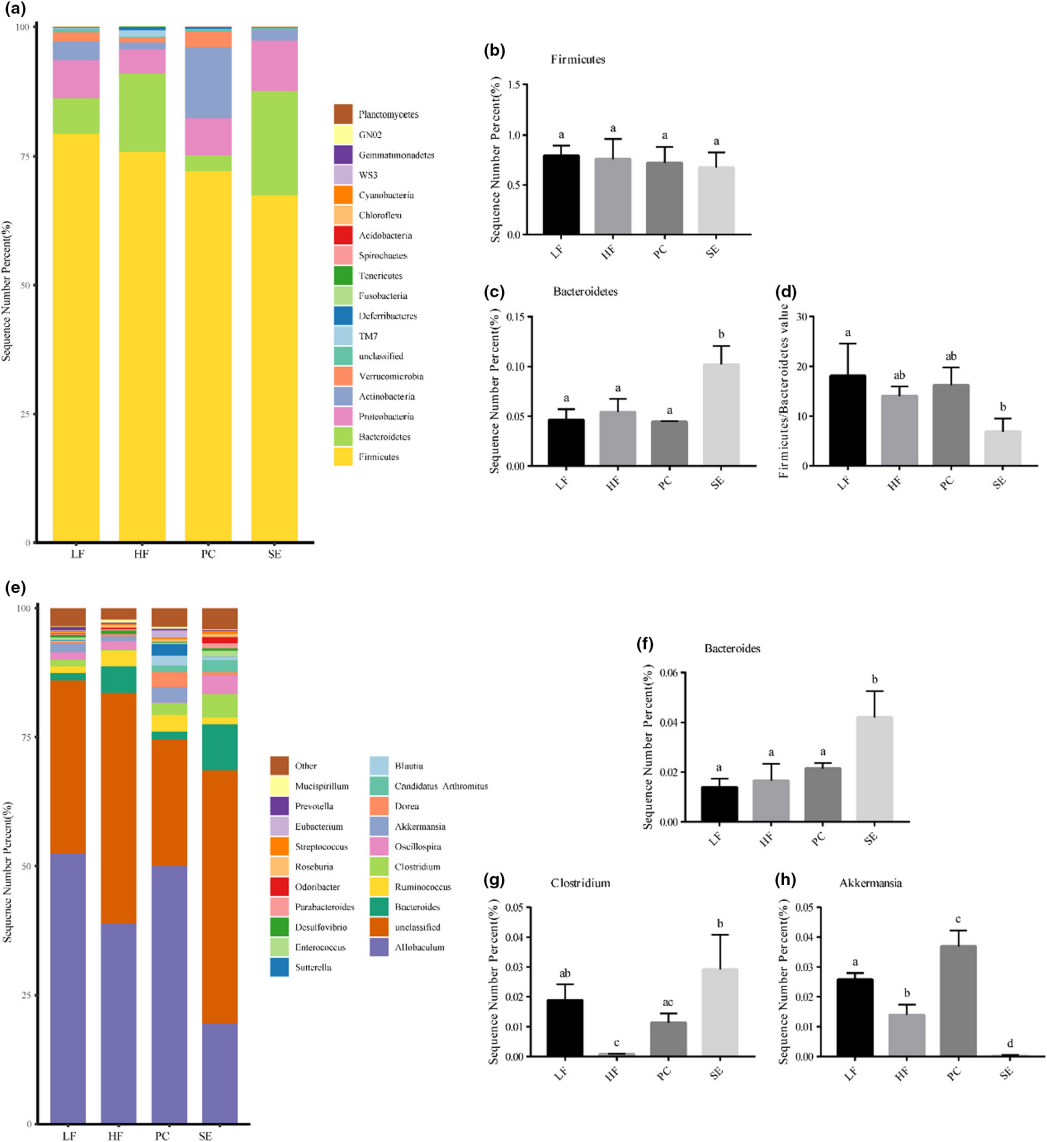
Effects of liposoluble components of highland barley grains on intestinal microflora level and genus level of high‐fat diet mice. (a) Relative abundance distribution at the level of intestinal microflora. (b) *Firmicutes*. (c) *Bacteroidetes*. (d) *Firmicutes/Bacteroidetes*. (e) Relative abundance distribution of intestinal flora at the genus level. (f) *Bacteroides*. (g) *Clostridium*. (h) *Akkermansia*.

The intestinal flora of mice in the LF group, HF group, PC group, and SE group mainly included *Firmicutes*, *Bacteroidetes*, *Proteobacteria*, *Actinobacteria*, and *Verrucomicrobia* (Figure 4a). There was no significant difference between the groups for *Firmicutes*. The abundance of *Bacteroidetes* was significantly increased in the SE group. In addition, the F/B ratio of the SE group was significantly lower than that of the HF group (Figure 4b–d).

At the genus level, the composition of intestinal microorganisms in each group mainly included *Allobaculum*, *Bacteroides*, *Ruminococcus*, and *Clostridium*, among which *Allobaculum* and *Bacteroides* were the dominant flora (Figure 4e). Compared with the HF group, the SE group showed an increased relative abundance of *Bacteroides*, *Clostridium*, and other flora. *Akkermansia* was also found at the genus level (Figure 4f–h). *Akkermansia* is an important beneficial bacterium in the intestine. However, *Akkermansia* showed no increase in high‐fat diet‐fed mice after they fed on the liposoluble ingredients, possibly because *Akkermansia* is not a target of the liposoluble components. The above results show that the liposoluble components of highland barley spent grains can regulate the structure of the intestinal flora and have the potential to improve lipid metabolism and related symptoms in mice fed a high‐fat diet. The specific effect and the mechanism involved need to be further verified by additional experiments.

### 3.7 Transcriptomic analysis of mouse liver

#### 3.7.1 Transcriptome sequencing data and comparative analysis

We sequenced the liver transcriptomes of the LF group, HF group, and SE group mice. The comparison of the transcriptome sequencing results with the reference genome revealed a total of 406,102,138 original reads, and 404,256,830 clean reads were obtained after quality control (Table 5). The Q20 values were >97%, and the Q30 values are >93%. This shows that the quantity and quality of the sequencing data met the requirements for subsequent analysis.

**TABLE 5:** Sample sequencing quality and sequence alignment.

Samples	Raw reads	Clean reads	Q20 (%)	Q30 (%)	GC content (%)
LF	45,041,962	44,860,392	97.88	93.53	46.34
LF	58,543,386	58,281,178	97.96	93.8	47.11
LF	44,204,164	43,989,980	97.85	93.57	45.78
HF	43,149,068	42,951,754	97.95	93.81	47.02
HF	42,461,506	42,274,566	97.83	93.47	45.94
HF	44,182,162	43,980,262	97.91	93.71	46.96
SE	43,534,742	43,319,916	97.79	93.34	44.78
SE	41,793,028	41,611,460	97.71	93.08	44.73
SE	43,192,120	42,987,322	97.83	93.44	45.08

#### 3.7.2 Analysis of expressed genes and differentially expressed genes (DEGs) between samples

**FIGURE 5:**
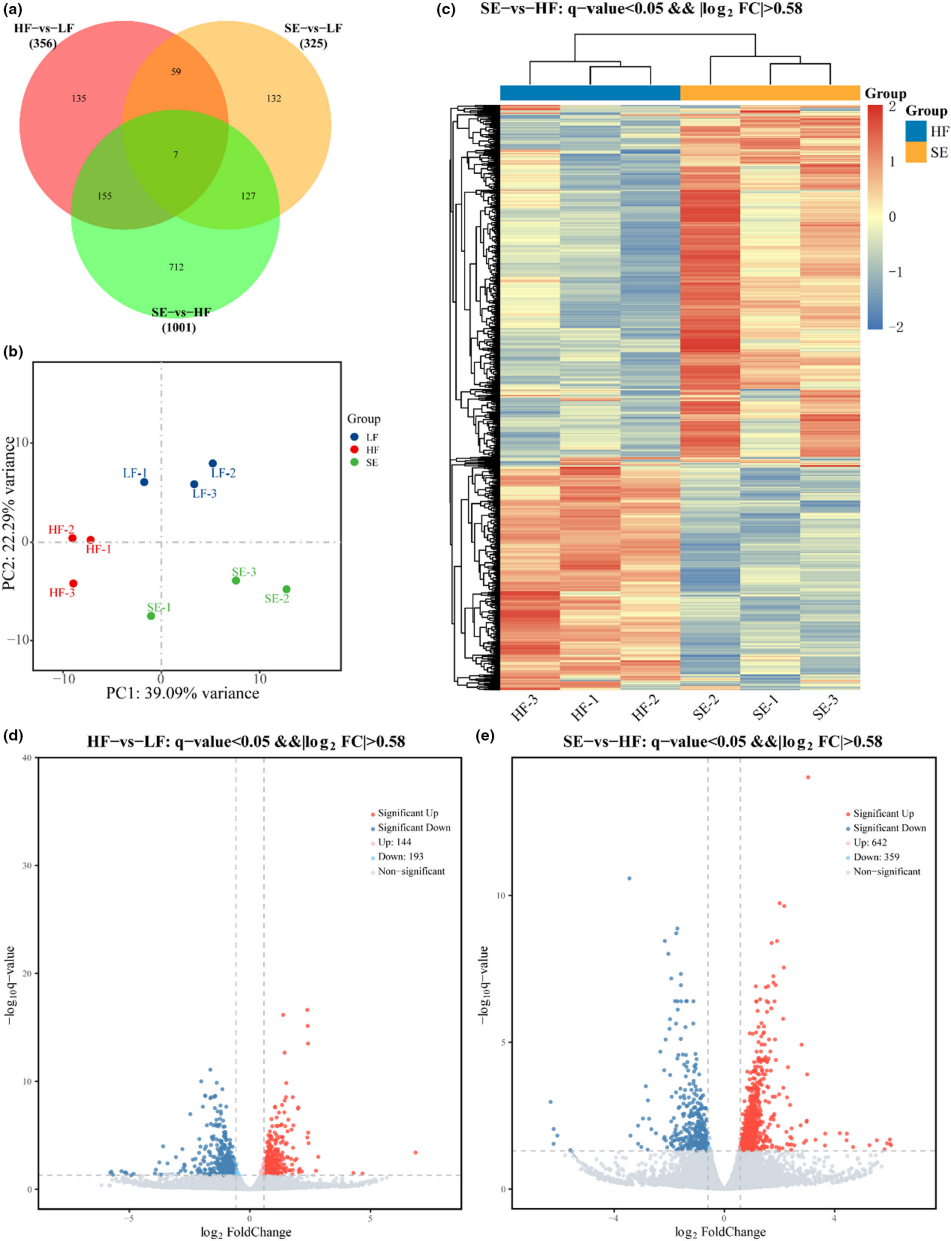
(a) Venn diagram. (b) principal component analysis. (c) cluster heat map of differentially expressed genes in SE versus HF. (d) HF versus LF liver differentially expressed genes. (e) SE versus HF liver differentially expressed genes.

The unique and common differentially expressed genes among the different controls and HF versus LF, SE versus LF, and SE versus HF are shown in Venn diagrams (Figure 5a). In the HF versus LF, SE versus LF, and SE versus HF comparisons, there were 7 common genes and 135, 132, and 712 unique genes, respectively. In addition, according to the gene expression levels among different samples, principal component analysis was performed, and the three groups were significantly aggregated and showed significant separation from each other, indicating that the low‐fat diet and the liposoluble components of highland barley grains could significantly affect the expression of genes in the mouse liver (Figure 5b). Specifically, there was clear separation between the LF and HF groups, indicating that the high‐fat diet significantly altered the gene expression profile of the mouse liver. The significant separation between the HF and SE groups after feeding on the lipid‐soluble components of distillery grains suggests that the lipid‐soluble component intervention can significantly reverse some of the gene expression changes induced by a high‐fat diet. The cluster heatmap of differentially expressed genes in the SE and HF groups (Figure 5c) showed that samples from the same group appeared in the same cluster, and the samples of different groups were clearly distinguished. The differences between the SE and HF groups indicated that different dietary treatments resulted in significant differences in the regulation of differentially expressed genes. Figure 5d,e shows the differentially expressed genes among the LF, HF, and SE groups (*p* < .05 and FC ≥ 1.5). A total of 356 genes were significantly differentially expressed between HF and LF mice. A total of 1001 genes were significantly different between SE and HF mice.

#### 3.7.3 Functional annotation and gene set enrichment analysis

**FIGURE 6:**
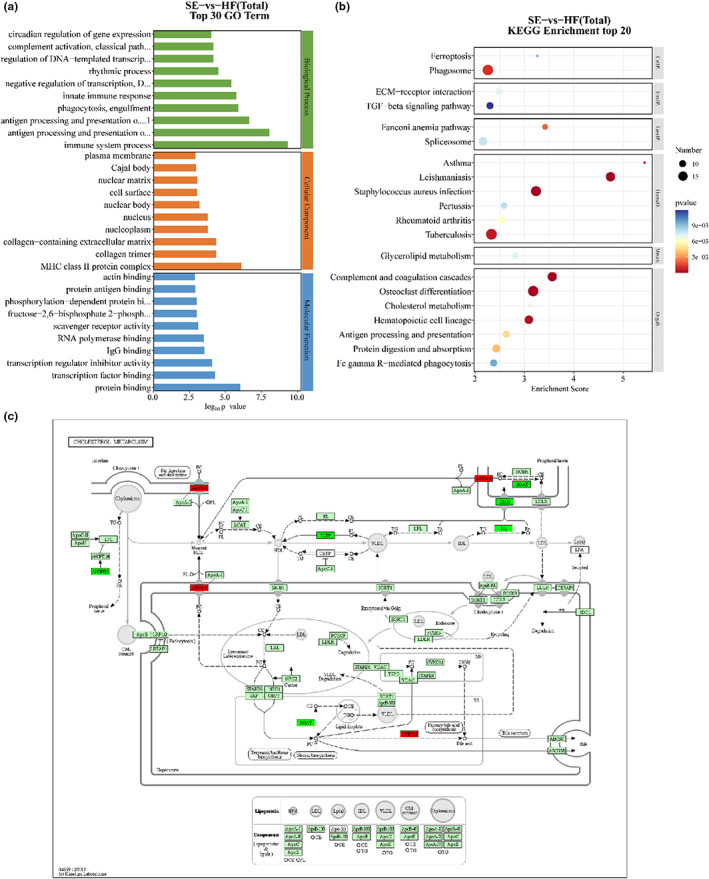
GO and KEGG enrichment analysis results (a) GO enrichment analysis of differentially expressed genes in SE versus HF. (b) KEGG signaling pathway enrichment analysis of differentially expressed genes (SE vs. HF). (c) Cholesterol metabolic pathway map.

The GO database was used to annotate differentially expressed genes in three categories: biological process, molecular function, and cell component (Figure 6a). The top three terms identified were immune system process (56 genes, biological process), MHC class II protein complex (6 genes, cellular component), and protein binding (312 genes, molecular function). The KEGG database is a resource for understanding the high‐level functions and roles of biological systems such as cells, tissues, and ecosystems based on molecular‐level information. KEGG enrichment analysis is an effective method for elucidating the biological functions of DEGs. According to the identified KEGG pathways, the gene sets were evaluated and grouped into six main types: human diseases, organic systems, cellular processes, environmental information processing, genetic information processing, and metabolism (Figure 6b). Cholesterol metabolism (7 genes, organismal systems), glycerolipid metabolism (8 genes, organismal systems), and the TGF‐beta signaling pathway (10 genes, environmental information processing) were found to be correlated with lipid metabolism. The results of enrichment analysis showed that SE may regulate the physiological indexes, metabolism, inflammatory responses, and biological processes of mice mainly through the cholesterol metabolism and glycerolipid metabolism pathways.

#### 3.7.4 Cholesterol metabolic pathway analysis

According to the transcriptome analysis, we found that the lipid‐soluble components of distillery grains affected the expression of various genes in high‐fat diet‐fed mice, which could mediate the normal functions of various pathways and eventually lead to the abnormal expression of various proteins. The identified pathways closely associated with fat metabolism were glycerolipid metabolism and cholesterol metabolism, among which the differentially expressed genes enriched in cholesterol metabolism pathways included CD36, ANGPTL8, SOAT1, LIPC, ABCA1, CYP7A1, and PLTP. These results suggest that the lipid‐soluble components of distillers' grains regulate lipid metabolism in high‐fat diet‐fed mice, which may be related to the expression of these genes. Cholesterol metabolism is related not only to lipid metabolism but also to cholesterol metabolism, which was the most significant metabolic pathway, so the cholesterol metabolism pathway is mainly discussed below. In addition to these key differentially expressed genes, we also analyzed the protein translation of differentially expressed genes that may be involved in the entire pathway of cholesterol metabolism (Figure 6c). The light green background of the cholesterol metabolism pathway represents the proteins involved in the various steps of the pathway. Differentially expressed genes are mapped to the KEGG pathway. Nodes in the red border represent the expression of related proteins whose levels were significantly upregulated by differentially expressed genes, and dark green represents the expression of related proteins whose expression was significantly downregulated by differentially expressed genes. The translation of proteins at a node may involve multiple differentially expressed genes. The liposoluble components of highland barley grains can inhibit the mRNA expression of ANGPTL8, CD36, PLTP, and SOAT1, promote the mRNA expression of CYP7A1 and ABCA1, and play a role in liver cholesterol metabolism by regulating the three pathways of cholesterol decomposition, reverse transport, and absorption. That is, the CYP7A1 gene is regulated to promote the decomposition of cholesterol, the ABCA1, CD36, and PLTP genes are regulated to promote the reverse transport of cholesterol, and the SOAT1 gene is regulated to inhibit the absorption of cholesterol.

## 4 DISCUSSION

Serum biochemical indexes of animals can reflect the overall metabolic status of the organism to a certain extent, and abnormal changes in biochemical indexes can indicate that the functional status of a certain tissue or organ has changed. A high concentration of T‐CHO in blood will lead to the deposition of lipid substances on blood vessel walls+, which will cause the blockage and stenosis of blood vessels, resulting in obstructed blood flow and likely causing atherosclerosis, hyperlipidemia, and other cardiovascular diseases (He et al., 2020). LDL‐C plays an important role in atherosclerosis, and its content is positively correlated with the risk of cardiovascular disease. Although LDL‐C can be used as a predictor of atherosclerosis, the LDL‐C/HDL‐C ratio can better reflect the degree of atherosclerosis and has more clinical significance, and it is an important indicator of atherosclerosis (Du et al., 2020). In this study, the intervention of feeding mice spent grain liposoluble components had an improvement effect on dyslipidemia, resulting in decreases in the levels of T‐CHO, TG, and LDL‐C and an increase the level of HDL‐C in high‐fat diet‐fed mice. ALT and AST levels are important indicators for evaluating liver function. When liver tissue is damaged, the permeability of the cell membrane increases, and ALT and AST in the liver cytoplasm are released into the blood, leading to increases in liver and serum transaminase levels (Norris et al., 2017). The results showed that the liposoluble components of lees significantly reduced the liver aspartate aminotransferase (AST) levels of high‐fat diet‐fed mice (*p* < .05). As an important product of lipid peroxidation, MDA is an indirect indicator for evaluating the generation of free radicals and the occurrence of lipid peroxidation, and changes in its content reflect the degree of oxidative damage (Fang et al., 2019). As an antioxidant, GSH can protect cells from oxidative damage and detoxify cells. When the GSH content is reduced or even depleted, it indicates that the body has suffered oxidative damage, which can cause toxic effects in serious cases. Therefore, GSH is also an important indicator of oxidative damage (Li et al., 2019). Under normal physiological conditions, the antioxidant enzyme SOD plays a role in scavenging oxygen‐free radicals, and a decrease in its content indicates that the body's ability to eliminate oxygen free radicals is weakened (Jiang et al., 2016). GSH‐Px is an enzyme that plays an important role in the reduction of peroxide and can reduce the damage to the body caused by peroxide. H_2_O_2_ generated in the body is decomposed or utilized by CAT to avoid oxidative damage (Wu et al., 2019). The experimental results showed that the liposoluble components of spent grains also had significant effects on the liver antioxidant indexes of mice, and intervention with liposoluble components significantly increased the levels of SOD, CAT, GSH, and GSH‐Px in the livers of mice (*p* < .05) and significantly reduced the level of MDA (*p* < .05). The effect of spent grain liposoluble components on improving serum and liver conditions may be related to the compositions of fatty acids and phytosterols. Chen et al. (2019) showed that different compositions and ratios of polyunsaturated fatty acids could prevent atherosclerosis (As) in mice, reduce serum TG, T‐CHO, and LDL‐C, effectively inhibit the formation of aortic As plaques, and increase MUFA and that PUFA intake exerted an antiatherosclerotic effect. In addition, phytosterols, the liposoluble components of spent grains, also have antioxidant and cholesterol‐lowering effects. Zhang et al. (2019) extracted pumpkin seed sterol from pumpkin seed oil and fed it to SD rats intragastrically for 35 days. Compared with the blank control, the activity levels of GSH‐Px, SOD, T‐AOC, and MDA in the experimental group were significantly increased, indicating that pumpkin seed sterol enhanced the antioxidant capacity of SD rats.

In our experiment, it was found that the dominant flora at the phylum level were *Firmicutes*, *Bacteroidetes*, *Proteobacteria*, *Actinobacteria*, and *Verrucomicrobia*. Studies have shown that an increase in the *Firmicutes/Bacteroidetes* ratio is closely related to the occurrence and development of various diseases, such as inflammatory bowel disease (Li et al., 2022) and obesity (Demirci et al., 2020). In addition, the abundance of *Bacteroidetes* was previously shown to be lower in a type II diabetes group, the abundance of *Firmicutes/Bacteroidetes* was higher, and the abundance of *Proteobacteria* and *Actinobacteria* was higher (Sedighi et al., 2017). However, the F/B ratio and *Proteobacteria* and *Actinomycetes* counts of mice fed a high‐fat diet were decreased by the liposoluble components of distillers' grains, suggesting that the liposoluble components could improve resistance to disease in mice to a certain extent. Other studies have shown that *Bacteroidetes* can inhibit the colonization of the intestinal flora by harmful bacteria and enhance the body's resistance to viruses, which is crucial to ensure intestinal health (Sequeira et al., 2020). Compared with the HF group, the F/B value of the SE group was significantly decreased, and *Bacteroidetes* abundance was significantly increased, suggesting that the liposoluble components of highland barley lees could regulate the intestinal microflora structure of high‐fat diet‐fed mice.

*Allobaculum*, *Bacteroides*, *Ruminococcus*, and *Clostridium* were the dominant flora at the genus level. Compared with the HF group, the SE group showed increased relative abundances of *Bacteroides* and *Clostridium*. Studies have shown that species of *Bacteroidetes* have a variety of probiotic effects and can help the host decompose and utilize polysaccharides and produce short‐chain fatty acids to provide energy for the host (Xiang et al., 2021). In addition, these bacteria play an important role in promoting intestinal mucosal angiogenesis and immune system development (Mcilroy et al., 2018). *Clostridium* indirectly promotes intestinal peristalsis by stimulating intestinal cells to enhance the release of the neurotransmitter 5‐hydroxytryptamine (Yano et al., 2015). These results indicate that the liposoluble components of highland barley grains can regulate the intestinal microflora structure and have the potential to improve lipid metabolism and related diseases in high‐fat diet‐fed mice.

KEGG pathway enrichment analysis in the SE versus HF groups after feeding on liposoluble components of barley grains showed had multiple pathways related to fat metabolism, mainly glycerolipid metabolism, and cholesterol metabolism. In the cholesterol metabolism pathway, the main genes showing significant differences were CD36, ANGPTL8, SOAT1, LIPC, ABCA1, CYP7A1, and PLTP. ABCA1 can promote the efflux of phospholipids from cells and then combine with apoA‐I to form disc HDL, which can subsequently produce mature HDL rich in cholesterol esters under the action of LCAT, initiating cholesterol reversal. If overexpressed, it can cause cholesterol outflow and promote HDL‐C production. A phospholipid transporter (PLTP) can transfer cholesterol carried by mature HDL to LDL, and some of the LDL is then oxidized to ox‐LDL and returned to the cholesterol synthesis pathway in the liver by CD36. CD36 is a member of the class B scavenger receptor (SREBP) family, which is associated with fatty acid transport and cholesterol reversal transport. Binding with long‐chain free fatty acid ligands can mediate lipid uptake and cell decay (Rada et al., 2020). CYP7A1 is a key rate‐limiting enzyme in the decomposition pathway, and the expression level of CYP7Al in the liver can reflect the degree of cholesterol decomposition and excretion and the rate of bile acid synthesis (Duan et al., 2019). SOAT1 is a key enzyme in cholesterol ester biosynthesis (Volkmar et al., 2019), which is essential for maintaining intracellular lipid metabolism homeostasis. ANGPTL8 gene knockout can inhibit the accumulation of FREE fatty acid‐induced TG in liver cells (García‐Monzón et al., 2018). In this study, it was found that the mRNA expression levels of ANGPTL8, CD36, PLTP, and SOAT were lower in the SE group, while the mRNA expression levels of CYP7A1 and ABCA1 were higher. These results suggest that the cholesterol metabolism pathway mediated by ANGPTL8, CD36, PLTP, SOAT, CYP7A1, and ABCA1 plays a role in lipid metabolism and participates in lipid metabolism and other biological processes in high‐fat diet‐fed mice. The liposoluble components of highland barley grains can reduce cholesterol by reducing the mRNA expression of ANGPTL8, CD36, PLTP, and SOAT, increasing the mRNA expression of CYP7A1 and ABCA1, promoting the transport of cholesterol, inhibiting the absorption of cholesterol, and accelerating the transformation of cholesterol into bile acid, thus reducing cholesterol levels. Ultimately, these liposoluble components can improve cholesterol metabolism in the serum and liver of high‐fat diet‐fed mice.

## 5 CONCLUSION

In this study, it was found that the liposoluble components of highland barley grains could significantly improve the serum and liver indices and intestinal microflora of mice on a high‐fat diet, suggesting that the liposoluble components of highland barley grains could significantly improve the antioxidant capacity and the species richness and diversity of the intestinal microflora of mice fed a high‐fat diet. Gene‐level transcriptomic analysis also showed that the effects of liposoluble components on genes related to fat metabolism in high‐fat diet‐fed mice were reflected in the improvement of the lipid‐lowering (e.g., cholesterol‐lowering) ability. Therefore, the liposoluble components of spent grains can provide a reference for regulating serum and liver indexes, fat metabolism, and fat loss in mice fed a high‐fat diet and can provide a theoretical basis for research on weight loss and fat reduction associated with the consumption of highland barley grain.

## CONFLICT OF INTEREST STATEMENT

The authors declared no potential conflicts of interest with respect to the research, authorship, and publication of this article. All authors disclosed no relevant relationships.

## Data Availability

Data are available with the corresponding author of this publication upon reasonable request.
